# Establishment of the *Q*_y_ Absorption Spectrum of Chlorophyll *a* Extending to Near-Infrared

**DOI:** 10.3390/molecules25173796

**Published:** 2020-08-20

**Authors:** Kristjan Leiger, Juha Matti Linnanto, Arvi Freiberg

**Affiliations:** 1 Institute of Physics, University of Tartu, W. Ostwaldi 1, 51011 Tartu, Estonia; kleiger@ut.ee (K.L.); juha.matti.linnanto@ut.ee (J.M.L.); 2 Institute of Molecular and Cell Biology, University of Tartu, Riia 23, 51014 Tartu, Estonia; 3 Estonian Academy of Sciences, Kohtu 6, 10130 Tallinn, Estonia

**Keywords:** photosynthesis, chlorophyll *a*, *Q*_y_ state, near-infrared absorption tail, vibronic transitions, fluorescence lifetime, anti-Stokes excitation spectroscopy, TD-DFT CAM-B3LYP calculations

## Abstract

A weak absorption tail related to the *Q*_y_ singlet electronic transition of solvated chlorophyll *a* is discovered using sensitive anti-Stokes fluorescence excitation spectroscopy. The quasi-exponentially decreasing tail was, at ambient temperature, readily observable as far as −2400 cm^−1^ from the absorption peak and at relative intensity of 10^−7^. The tail also weakened rapidly upon cooling the sample, implying its basic thermally activated nature. The shape of the spectrum as well as its temperature dependence were qualitatively well reproduced by quantum chemical calculations involving the pigment intramolecular vibrational modes, their overtones, and pairwise combination modes, but no intermolecular/solvent modes. A similar tail was observed earlier in the case of bacteriochlorophyll *a*, suggesting generality of this phenomenon. Long vibronic red tails are, thus, expected to exist in all pigments of light-harvesting relevance at physiological temperatures.

## 1. Introduction

Chlorophylls are the most abundantly found pigments on Earth, facilitating the important functions of absorbing solar energy and electron transport in photosynthetic organisms such as plants, algae, and bacteria. The distinct role of these molecules in the utmost processes of nature relies on their unique optical and redox properties. It is, thus, expected that every aspect of their optical spectra is already thoroughly studied. This is not quite the case. Naturally, the basic absorption–emission spectral data have been around for decades—see [1] for comprehensive reviews—yet, there are many details critical for understanding, e.g., coherent exciton transport in photosystems, a focus of many recent works [2,3,4,5,6,7,8].

Pertinent to exciton dynamics is both the ground-state and the excited-state reorganization energy, which can be measured and partitioned into individual vibrational components using modern high-resolution techniques [9,10,11,12,13]. The results obtained for the lowest singlet excited electronic state *Q*_y_ of chlorophyll *a* (Chl *a*) [14,15], bacteriochlorophyll *a* (BChl *a*) [16], and pheophytin *a* [17] indicate fundamental absorption–emission asymmetry, imposing a shift on the long-standing paradigm, which assumes that absorption and emission spectra of chlorophylls associated with the *Q*_y_ state are mirror-symmetric (or nearly so). That is, after appropriate normalization and reflection of (say) the emission spectrum about its origin, the two spectra are identical. While essential for straightforward quantum dynamics calculations [18], the observation of high-resolution spectra showed that the reality is different. Many of the intense modes in either absorption or emission are just not seen at all in the other spectrum [14,15,16,17,19,20], suggesting the possibility of serious failure for symmetry-based quantum dynamics models of exciton and electron transfer. Furthermore, the asymmetry is shown to be environment-dependent, allowing, in principle, metal chelation and changes to nearby residues and solvent location to modulate transport in a protein environment.

Another spectral property pertinent to functionality of chlorophylls, so far neither adequately recognized nor effectively addressed, is the shape of the long-wavelength tail of the *Q*_y_ absorption band. However, being related to the well-documented red drop of the photosynthesis quantum yield [21,22,23], its significance would be difficult to overestimate. Moreover, as reviewed in [24], additional low-energy states have been discovered in many systems with obscure origins and significance. Since low-energy states are expected to be critical to function, these observations seemingly bypassing the established higher-energy mechanisms pose important challenges. Many recent models relate the low-energy states with charge transfer states [25].

The rather large reach of absorption of photosynthetic pigments to the red has caught our attention before [26], when studying circular, BChl *a*-containing light-harvesting pigment–protein complexes from purple bacteria. Pigments in these complexes are closely packed, facilitating delocalization of the BChl *a* excited states, even at high temperatures [27,28], over a number of molecules. The resulting exciton spectra characterize the pigment–protein complex as a whole. However, to yield an understanding about the rich assortment of interactions taking place between the pigment molecules as well as between the pigments and their immediate protein surroundings [29], one has to study the pigment molecules individually, and as much as possible, in impartial environments. The basic information gained from such measurements also facilitates performing numerical simulations to support the data obtained from studies of native photosystems.

In a previous work [30], we measured the absorption red tail of BChl *a* molecules dissolved in triethylamine (TEA). The tail turned out to decrease roughly exponentially toward near-infrared, being at ambient temperature well measurable up to ~2400 cm^−1^ from the *Q*_y_ band maximum. The long tailing was explained by absorption from thermally excited intramolecular vibrational levels of BChl *a*, including the overtones of fundamental modes.

Herein, we set out to investigate the red absorption tail of Chl *a*. To maintain compatibility with our previous measurements, TEA solvent was used and the measurements were performed by means of an anti-Stokes excitation spectroscopy technique, explained in detail in the Materials and Methods section. TEA is convenient because in this solvent, the coordination state of the Chl *a* central magnesium is five-fold, similar to native systems, and Chl *a* is relatively resistant to the light-induced oxidation, a welcomed feature under the intense laser excitation applied. Besides, the TEA molecule is fairly compact, hence, computationally well manageable. As was convincingly demonstrated in [30], application of excitation spectroscopy in the measurements of weak absorption tails of fluorescing samples is superior compared with absorption spectroscopy due to the much higher sensitivity of this method. Recording absorbance via fluorescence also adds specific selectivity to the method.

## 2. Results and Discussion

**Figure 1:**
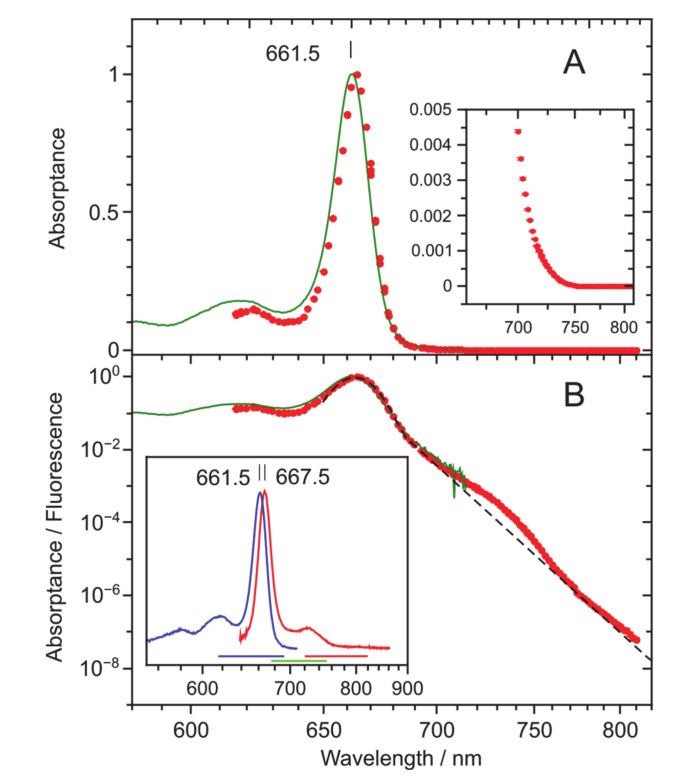
The normalized *Q*_y_ absorptance (dark green solid line) and fluorescence excitation (red symbols) spectra of Chl *a* in TEA measured at ambient temperature of 22 ± 1 °C (295 ± 1K). The signal intensity scale is linear in part Figure 1A and logarithmic in part Figure 1B to emphasize the peak and tail regions of the spectra, respectively. Note also that here and henceforth, a reciprocal (linear in energy) wavelength scale is used. Shown also in Figure 1A is the 200-fold amplified red part of the spectrum and in panel B, an approximate partition of the *Q*_y_ bandshape into Gaussian origin and quasi-exponentially decaying tail parts (dashed black line). The insert of Figure 1B shows the conventional absorption (blue) and fluorescence (red) spectra of Chl *a* in the same solvent. Horizontal colored lines indicate the excitation ranges in red, blue, and intermediate (green) spectral regions, while the filtered fluorescence is registered over complementary ranges (see Materials and Methods).

Figure 1 presents the *Q*_y_ absorptance (1−transmission) and fluorescence excitation spectra of Chl *a* dissolved in TEA with a concentration of ~6∙10^−6^ M. The two representations of Figure 1 with ordinary linear (A) and logarithmic (B) intensity scales serve to highlight distinct ranges of the *Q*_y_ spectrum: its strong band origin (A) and weak tail (B).

Reasonable within the experimental resolution of 1.5 nm, a spectral overlap between the measured absorptance and fluorescence excitation spectra is observable between 615 and 715 nm. This range is, from the blue side, limited by the tuning range of the available lasers required for measurements of fluorescence excitation spectra and from the red side, by low sensitivity of absorption measurements. The several orders of magnitude higher sensitivity of the measurements of excitation spectra in comparison with that of absorption/absorptance is very clearly demonstrated in the semi-logarithmic plot of Figure 1B.

From Figure 1B, one can also perceive that the absorption/absorptance band shape of Chl *a* can be broadly described by a combination of central Gaussian and red-side exponentially decreasing parts (drawn by dashed line). Similar general dependence was formerly observed for BChl *a* [30]. However, as it will be elaborated below in more detail, compared to BChl *a*, the Gaussian part of Chl *a* is significantly narrower and, most remarkably, there is a prominent “hunch” apparent between 700 and 750 nm. Although a deviation from exponentiality at similar energetic distance from the *Q*_y_ maximum was also noted in BChl *a*, it was rather minor.

In order to learn about the physical origin of the quasi-exponentially decreasing red tail, its temperature dependence was studied by recording the spectra at several temperatures between the ambient temperature of 295 and 220 K. The latter temperature, which is reasonably higher than the freezing temperature of TEA (158 K [31]) was chosen to avoid any sample solidification effects on the data. A reasoning behind the temperature-dependence measurements is that if the long tail is related to the absorption from the hot (thermally populated) vibrational levels of the ground electronic state, as suggested in the case of BChl *a* [30]; the tail absorbance is, according to the Boltzmann distribution, bound to diminish exponentially with lowering the temperature.

**Figure 2:**
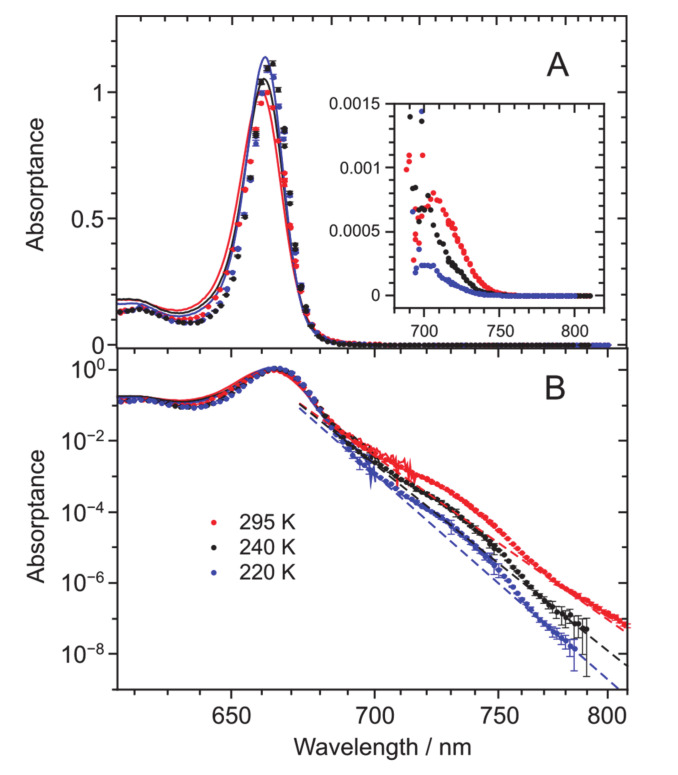
Integral intensity-normalized absorptance (solid lines) and fluorescence excitation (discrete symbols marked with the experimental uncertainty) spectra recorded at different temperatures indicated and presented in linear (**A**) and half-logarithmic (**B**) scale. Shown in the inset of **A** are the amplified “hunch” spectra, i.e., differences between the actually measured excitation spectra and their approximate presentations as exponentially decaying tails depicted by dashed lines in **B**. Concentration of the samples about the same as in Figure 1.

Corresponding data are depicted in Figure 2. The spectra at three different temperatures (295, 240, and 220 K) presented so as to keep the integral intensity constant, qualitatively confirm the expected result. The tail absorbance decreases nearly exponentially with lowering the temperature. The absorptance maximum also grows upon lowering the temperature, simultaneously shifting to the red (Figure 2A). Whilst the shift has previously been ascribed to the increased polarity of the solvent upon cooling [32], the growth is a combined effect of spectral narrowing and an increase in the *Q*_y_ origin absorbance. About a 10% increase in the total absorbance integrated between 640 and 720 nm was observed when the temperature was decreased from 295 to 220 K (data not shown).

As additionally seen in Figure 2A, there appears to be a small (compared to the recognized experimental uncertainty), yet systematic deviation between the experimental absorptance and excitation spectra, the blue side of the excitation spectrum falling down faster than the absorptance spectrum. As a result, excitation spectra at all temperatures seem slightly narrower and red shifted with respect to absorptance spectra; its effect on red side of the origin spectra is minor, though. The reason for this discrepancy is currently unclear, calling for additional investigations. One may suspect photoselection by partially polarized laser excitation in fluorescence excitation measurements, conceivable due to near mixing between the *Q*_y_ and *Q*_x_ states [14,15] of Chl *a*. Yet, a similar effect was observed in BChl *a* [30], where the *Q*_y_ and *Q*_x_ states are energetically well separated from each other.

To perceive the details of spectral structure of the “hunch” and its temperature dependence, the approximate exponentially decaying tails depicted in Figure 2B by dashed lines were subtracted from the actually measured excitation spectra as a background. The so obtained (amplified) difference spectra are shown in the inset of Figure 2A. Strong temperature dependence of the spectra in terms of both shape and intensity is evident as well as expected. The spectra, which seem to hold a clear-cut maximum (at ~705 nm) only at close to ambient temperatures, decrease rapidly upon cooling. This intensity loss (and the accompanying change of the spectrum) is expected because all the vibration states involved become depopulated upon lowering the temperature. The experimentally observed behavior is, thus, characteristic of the intrinsic, i.e., pertaining to individual Chl *a* molecules, origin of the “hunch” rather than of some external reason, such as impurity absorbance or aggregation of the pigment molecules.

**Figure 3:**
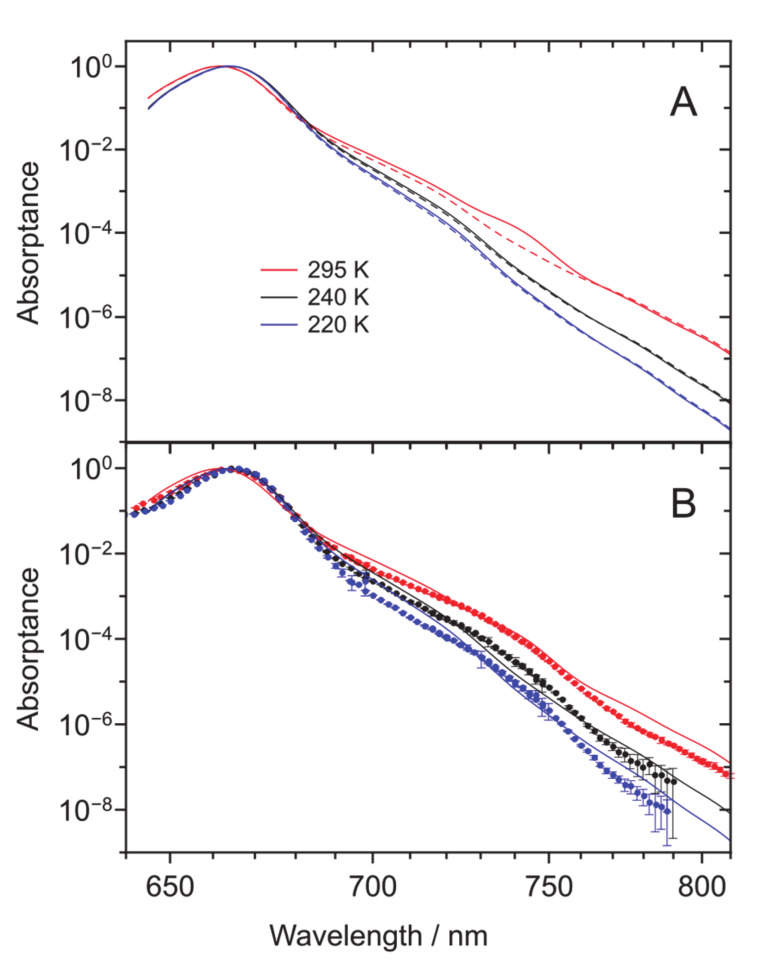
(**A**) Absorptance spectra of model Chl *a*: TEA calculated at different temperatures indicated. The calculated spectra involving either all fundamental modes and up to 20 overtones of each vibrational mode or the same together with *υ_i_* + *υ_j_* -type combinations of the modes, are presented by dashed or solid lines, respectively. (**B**) Comparison of the measured fluorescence excitation (discrete data) and calculated absorptance (solid lines) spectra. The spectra, normalized by the *Q*_y_ absorption maximum, are color-coded as in Figure 2.

Model calculations performed on a methylchlorophyllide *a* (MeChlide *a*): TEA 1:1 complex that mimics the minimalistic Chl *a*: TEA compound (see Materials and Methods) provide general support to this conjecture. Two sets of calculations were performed: one involving all fundamental modes plus up to 20 overtones of each vibrational mode and another, where additionally, the *υ_i_* + *υ_j_* -type combinations of the modes were included. Energy distributions of the vibrational modes accounted for in the calculations can be inspected in [33]. The respective spectra are in Figure 3A, drawn by dashed and solid lines. The more involved modeling has an effect only on the ambient temperature spectrum. The differences discovered at reduced temperatures are minor.

As shown in Figure 3B, the absorption spectra calculated at different temperatures readily reproduce both the Gaussian origin bands and the accompanying tails, which quasi-exponentially decay toward the near-infrared. However, in quantitative details, rather large discrepancies are observed between calculated and experimental spectra. Firstly, the calculated tails are slightly but systematic stronger than the experimental ones. Secondly, the “hunch” is poorly reproduced by the calculations. While the “hunch” appears to be present in the calculated ambient temperature spectrum at wavelengths shorter than 750 nm, it seems to be totally lost in low-temperature spectra. There might be multiple reasons selectively or in combination explaining these observations. However, without further investigations, we may only point here that the 1:1 MeChlide *a*: TEA complex used is hardly a perfect model for representing a real Chl *a* solvated in bulk TEA.

Provided the observed discrepancy in Figure 3 between the experimental and calculated “hunch” spectra around 700–750 nm, we next try to extra confirm its intrinsic Chl *a* origin by an experimental exclusion method. We start with noticing that one can probably discard traces of pheophytin *a*, an uncoordinated chromophore with missing magnesium in the pyrrole-containing ring, which has slightly red-shifted compared with Chl *a Q*_y_ absorption spectrum, from the list of potential discrepancy reasons. This is because the absorption–fluorescence spectra of Chl *a* and pheophytin *a* are rather different, i.e., easily distinguishable from each other (see, e.g., [17]), but most decisively, because of the inconsistency of this idea with the observed temperature dependence of the “hunch” structure. In the case of a trace, its spectral intensity would remain nearly constant with dropping temperature, in disagreement with the data in the inset of Figure 3A. We secondly remark that, like in our previous work [30], we made some test measurements where the TEA solvent used was initially dried by storing on molecular sieves (3 Å 1/16 by Wako) for at least 24 h prior to use. No change of the red tail was observed in comparison with the untreated solvent. Based on these data, inclusion of trace amounts of water as a possible factor influencing our main results was dismissed. Thirdly, dimers and oligomers of Chl *a* have been known to be responsible for the absorbance increase in the near-infrared close to 700 nm and even beyond, the precise position depending on the used solvent and temperature [32,34,35]. It has also been reported that the corresponding contribution should be more prominent in the case of relatively non-polar solvents, as otherwise, the solvent polarity would obstruct the dimerization/oligomerization process. TEA with its permanent molecular dipole moment of 0.7 D [36] certainly falls into this category. Aggregation is also favored—and in the case of non-polar solvents, only becomes possible—by cooling the sample. Yet, these are the effects we did not observe.

**Figure 4:**
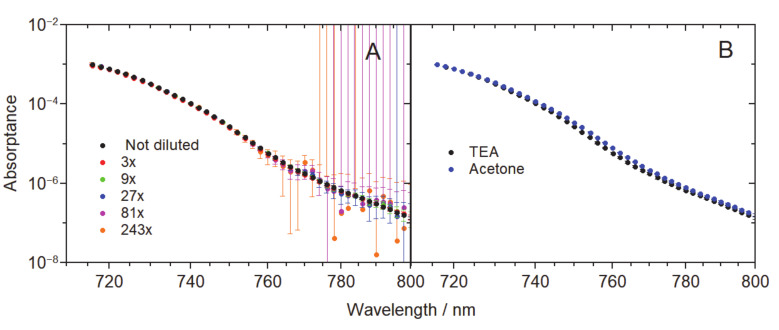
(**A**) Comparison of the fluorescence excitation spectra of Chl *a* in TEA with various degrees of dilution, as indicated in the legend. The initial concentration roughly corresponds to that in Figure 1. (**B**) Comparison of the excitation spectra of Chl *a* in TEA (black dots) and acetone (blue dots). In both panels, the vertical scale is normalized according to the *Q*_y_ peak at 661.5 nm equal to unity.

As a further test to assess the possible role of aggregation, we performed a series of measurements in dependence of the Chl *a* concentration and polarity of the solvent (Figure 4). Figure 4A depicts the red tail region of the excitation spectrum beginning from 716 nm for six different Chl *a* concentrations, starting from the same concentration as used in Figure 1 and Figure 2. Each subsequent dataset corresponds to three times diluted sample compared to the previous one. Hence, the whole series covers more than two orders of magnitude in terms of concentration. Yet, no difference exceeding the estimated experimental error margins could be detected. Since solvent polarity has been brought up in the literature as a critical factor suppressing the association of Chl *a* monomers [32], Figure 4B shows a comparison of the excitation spectra measured in two solvents—TEA and acetone—with contrasting dipole moments of 0.7 D in TEA and 2.5 D in acetone [36]. Although some discrepancy was observed between the measurements in different solvents, it is still minor compared with the difference between the real tail and its exponential approximation in the 700–750 nm region shown in the inset of Figure 2A.

In a final attempt to find evidence for the possible extrinsic nature of the “hunch”, we considered fluorescence kinetics measurements. The fluorescence lifetime in the Chl *a*: TEA system was measured by exciting and recording the fluorescence at various wavelengths across the whole *Q*_y_ absorption/fluorescence profile (data not shown). Upon non-resonant excitation at 430 nm, one and the same lifetime of 6.1 ± 0.3 ns of the mono-exponentially decaying fluorescence was obtained across the whole fluorescence profile between 640 and 760 nm; see inset of Figure 1. When exciting in the anti-Stokes region (to the red from the *Q*_y_ absorption maximum) between 730 and 760 nm, and recording at the fluorescence maximum around 668 nm, a decay time of 5.9 ± 0.1 ns was measured. This number matches the previous value within the experimental uncertainty. Taken together with other above facts, the observed independence of the fluorescence lifetime from either excitation or recording wavelength may serve as an additional argument for the “hunch” likely being an integral part of the *Q*_y_ absorption spectrum of the monomeric Chl *a* molecule.

**Figure 5:**
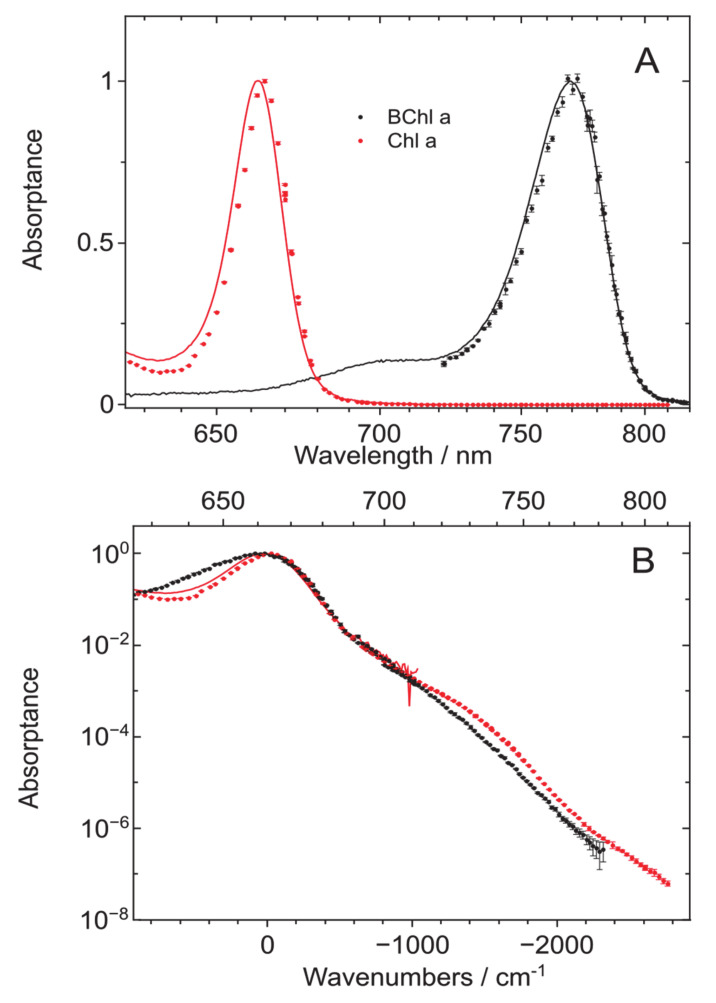
(**A**) Comparison of the *Q*_y_ peak-normalized absorptance (solid lines) and fluorescence excitation (discrete symbols) spectra from Chl *a* (red) and BChl *a* (black, data reproduced from reference [30]) in TEA at 295 K. (**B**) The same but with BChl *a* spectra blue-shifted to make the *Q*_y_ peaks of Chl *a* and BChl *a* coincide. Same axes as in Figure 1 and Figure 2 are applied, except for the bottom axis of Figure 5B, which represents the frequency shift in wavenumbers relative to the *Q*_y_ peak of Chl *a* and the corresponding blue-shifted peak of BChl *a*. See text for further explanations.

Relying upon this proof, we continue with the comparison between the absorptance and fluorescence excitation spectra of Chl *a* and BChl *a* (data reproduced from reference [30]) (Figure 5). The spectra shown in Figure 5A are measured at ambient temperature and plotted, as before, at reciprocal wavelength scale. In Figure 5B, the two spectra are overlapped by blue-shifting the BChl *a* spectrum.

The latter representation confirms rather similar extension and decay slope of the red spectral tails for both molecules. It also allows the identification of important differences between the two lineshapes as follows: Firstly, the Chl *a* spectrum possesses a significantly narrower *Q*_y_ origin band, the difference mainly originating from a more rapid drop of the blue part of the spectrum. Secondly, as already mentioned, the rather prominent “hunch” in the Chl *a* spectrum is but barely visible in the spectrum of BChl *a*.

**Figure 6:**
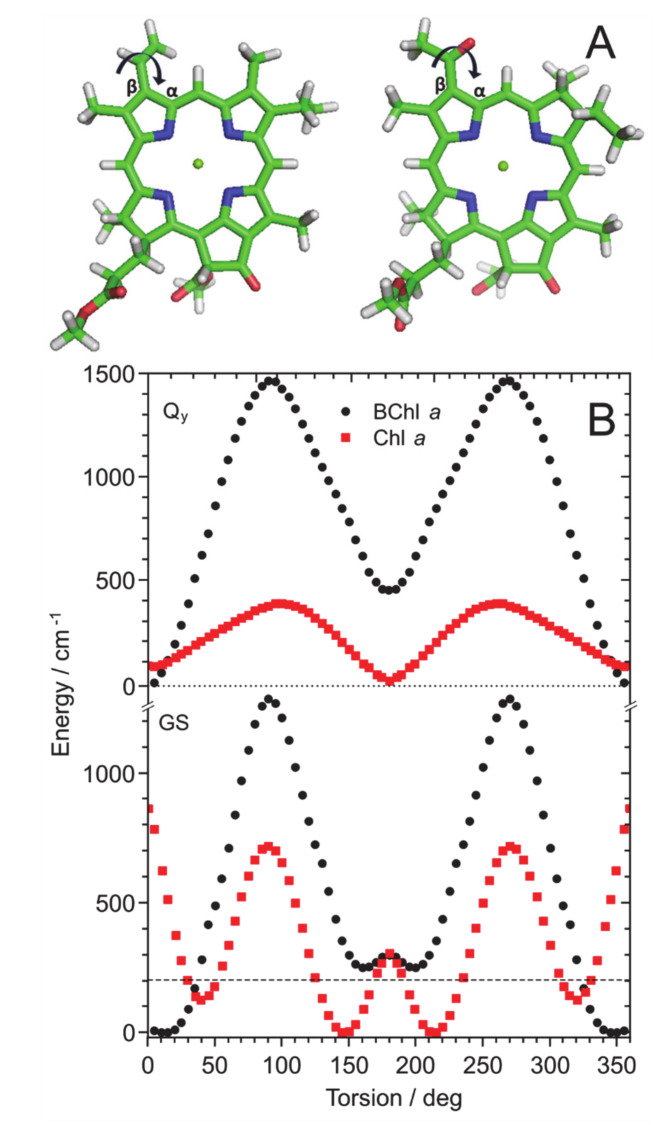
(**A**) The TD-DFT-CAM-B3LYP/6-31G (d, p) optimized molecular structures of MeChlide *a* (left) and MeBChlide *a* (right), molecules used as model structures for Chl *a* and BChl *a*, respectively. (**B**) Dependence of the ground-state (GS) and *Q*_y_ excited-state energies on the 3-vinyl/acetyl group torsion angle with respect to the cyclic tetrapyrrole π-plane (schematically indicated also in Figure 6A) in MeChlide *a* (red squares) and MeBChlide *a* (black dots) The horizontal dashed line shows the average thermal energy at ambient temperature: *k*_B_*T* = ~201.6 cm^−1^, where *k*_B_ is the Boltzmann constant and *T* is the absolute temperature.

It is known [37,38] that the ground- and *Q*_y_ excited-state energies of both Chl *a* and BChl *a* molecules are most sensitive to the orientation of the 3-vinyl/acetyl groups bound to the chlorin/bacteriochlorin skeleton, respectively, and that there are large energy barriers between the cis/trans isomers of the pigments. Armed with this previous knowledge, the dependence of the ground-state and *Q*_y_ excited-state energies on the torsion angle was calculated (Figure 6) using a TD-DFT-CAM-B3LYP/6-31G(d,p) method implemented in the Gaussian 16 program [39].

Since the equilibrium distribution of torsion configurations transforms directly to the *Q*_y_ band width, the much larger variation of the *Q*_y_ transition energy in BChl *a* compared to that in Chl *a*, seen in Figure 6, most probably qualitatively explains the greater bandwidth found in Figure 5 for the ensemble of BChl *a* molecules. As to the observed difference between the “hunches” in the spectra of Chl *a* and BChl *a*, it is much more difficult to evaluate theoretically. This is because according to our previous study [33], the PM7 method tends to underestimate vibrational intensities in the spectra of Chl *a* compared with those of BChl *a*.

## 3. Materials and Methods

Chlorophyll *a* from Sigma (St. Louis, MO, USA) was dissolved without further purification in TEA (also from Sigma) to the optical density of 0.5–1 cm^−1^ at the *Q*_y_ absorption maximum. The value 1 cm^−1^ corresponds to about 1.2 × 10^−5^ M (1.8 × 10^−6^ mol/mol) concentration of Chl *a* [40]. A quartz glass (Hellma, Müllheim, Germany) or homemade polymethylmethacrylate (PMMA) cuvette with 2 mm optical path length was used in experiments. The latter one was made of three 2-mm PMMA plates fused together with the central layer having two holes filled with sample and just solvent, respectively, so that any background emission not originating from the sample could easily be identified and subtracted as necessary. No contamination of the sample with Chl *a′* was detected using circular dichroism (data not shown), which is a reliable method for distinguishing Chl *a* epimers [41].

The measurements with spectral resolution of 1.5 nm were carried out using a home-built microspectroscopy system [42] composed of an Olympus IX-71 inverted microscope equipped with a 10x long-working-distance objective (NA = 0.28) (Mitutoyo, Kawasaki, Japan) and a Shamrock 303i spectrometer (Andor, Belfast, UK) together with an Andor spectroscopic camera iDus 420. Absorption/absorptance spectra were measured applying a standard tungsten halogen illumination lamp.

The fluorescence excitation sources were: a Spectra Physics (Mountain View, CA, USA) model 375 DCM dye laser (linewidth 0.5 cm^−1^) for the 615–700 nm range and a Model 3900S continuous wave Ti: Sapphire laser (tuning range 680–1020 nm, linewidth < 0.5 cm^−1^) for the 695–820 nm range. Both tunable lasers were pumped by an 8W Millennia Nd:YAG laser (all Spectra Physics). The excitation was delivered to the system via a 400 μm fiber (Thorlabs, Newton, NJ, USA) with collimators at each end and an additional set of two lenses in telescope arrangement was used to adjust the beam collimation and excitation spot size between 100 and 200 μm. The limits where the recorded signal level was in a linear spectroscopy (non-saturating) range were detected previously, and the excitation power applied was adjusted accordingly. Actual excitation power varied from 0.1 μW to 3.2 mW, depending on the wavelength and sample concentration. No sample bleaching effects were observed during the measurements.

The fluorescence decay kinetics were measured in transmission mode (i.e., exciting through the back side of the cuvette and collecting the signal from its front face) using a tunable femtosecond pulsed Ti: Sapphire laser (Mira Optima 900-F, Coherent, Santa Clara, CA, USA) with a pulse temporal/spectral width (defined as a full width at half-maximum, fwhm) of 100 fs/7 nm and the pulse repetition rate reduced to 3.8 MHz by applying a pulse picker. The fluorescence was recorded using a time-correlated single-photon counting system (SPC-150, Becker & Hickl GmbH, Berlin, Germany) equipped with an avalanche photodiode (ID 100–50, ID Quantique, Geneva, Switzerland). The kinetics were analyzed using Spectra Solve (Version 2.0, LASTEK Pty. Ltd, Adelaide, Australia) software and an experimentally determined temporal response function of the set-up.

The measurements at subnormal temperatures were performed in a microscope cryostat adapted for a diamond anvil cell (Oxford Instruments, Abingdon, UK and LOTO, Florence, Italy). Nitrogen from liquid nitrogen dewar was pumped through the cryostat for cooling. A Lakeshore thermal diode was placed near to the sample to get an accurate estimation of temperature and to switch the pump and heater on and off to maintain the temperature.

The fluorescence excitation spectra were recorded in three different wavelength regions according to the filters used to block the laser scattering: in the blue (615–690 nm, 700 and 750 nm longpass filters (Andor)), intermediate (670–750 nm, FES0650 filter (Thorlabs)), and red (720–820 nm, FES0700 filter (Thorlabs)) regions. The sample fluorescence was recorded over regions of about 700–920, 590–660, and 632–710 nm, in blue, intermediate, and red series, correspondingly; see inset of Figure 1B for reference of the noted wavelength ranges. The intermediate region was further divided into two regions where dye or the Ti: Sapphire laser could be used. The adjacent regions always had a mutually overlapping part, so they could all be subsequently combined. Relative intensities of the signal were determined by fitting the fluorescence shapes by linear scaling with a model shape obtained by averaging all measurements within a series. The resulting linear term was then divided by the reference power value recorded through the sample and corrected for the sample transmission, to get the excitation spectrum value at the corresponding wavelength. The measured excitation and absorption (recalculated to absorptance) spectra were finally fitted to match each other in the 630–700 nm range by adjusting the parameters so as to minimize their root mean square difference.

MeChlide *a* and methyl bacteriochlorophyllide *a* (MeBChlide *a*) molecules were used as model structures for Chl *a* and BChl *a*, respectively. Replacement of phytyl with methyl is not expected to have noticeable influences on the properties of interest herein, while its replacement with hydrogen can be influential [43,44,45]. The normal modes of vibrations were calculated for optimized electronic states of penta-coordinated MeChlide *a*/MeBChlide *a–*EA 1:1 complexes (β-TEA coordination complex according to IUPAC nomenclature of tetrapyrroles [46]) by using the MOPAC2016 program [47] with the semi-empirical PM7 method. A full configuration interaction approach with the four highest occupied and four lowest unoccupied molecular orbitals was used. Franck–Condon factors in harmonic oscillator approximation were evaluated by calculating overlap integrals between the vibrational eigenstates in the two optimized electronic states. The spectral calculations involved all possible transitions from the populated vibrational states of the electronic ground state in thermal equilibrium to all vibrational states of the lowest singlet excited state, up to 20 overtones of each vibrational mode and *υ_i_* + *υ_j_* -type combinations of the modes. Boltzmann distribution was used to generate initially populated vibrational state at experimental temperatures. The homogeneous line fwhm of 8 cm^−1^ was assigned for all vibronic/vibrational bands. A Gaussian random distribution with a fwhm of 300 cm^−1^ was applied to generate the inhomogeneously broadened *Q*_y_ spectral band shape suitable for comparison with the experiment.

## 4. Concluding Remarks

A weak near-infrared absorption tail related to the *Q*_y_ singlet electronic transition of Chl *a* solvated in organic solvents such as TEA or acetone was discovered using sensitive anti-Stokes fluorescence excitation spectroscopy. The quasi-exponential decrease of the tail with increasing wavelength was at ambient temperature reliably observable as far as ~2400 cm^−1^ apart from the *Q*_y_ transition peak. The tail also weakened rapidly upon cooling the sample, indicating its basic thermally activated nature. The fact that the shape of the spectrum as well as its temperature dependence is qualitatively well reproduced by quantum chemical calculations that involved just the pigment intramolecular modes, their overtones, and pairwise combination modes, but no intermolecular/solvent modes implies that the shape of the tail is primarily dependent on the properties of individual Chl *a* molecules. A similar tail was observed earlier in the case of BChl *a*, suggesting generality of this phenomenon. Long red tails are, thus, expected to exist in all related molecules.

The possible functional significance of the long red absorption tail of the main photosynthetic pigment remains to be analyzed. Given the low absorbance value, the light harvesting functionality in this spectral region is not very likely, except for some very special circumstances. It may, however, facilitate energy transfer through a network of antennas, as the energy transfer efficiency depends on spectral overlap between adjacent antenna complexes [33], (see [24] for a review of the possible roles of low-energy excited states in photosynthesis).

An exponentially decreasing tail of optical absorption has previously been observed also in Chl *a* containing Photosystem I (PSI) [48], being explained in the framework of the Urbach model. Urbach famously noticed that the absorption band shape of bulk crystalline solids can often be described as a combination of central Gaussian and red-side exponentially decreasing parts [49]. The model has since been generalized to hold other systems (see [50] for a review), but it has invariably been related with phonons (collective vibrations of the surroundings), not with local intramolecular vibrations of the involved pigments. Yet, the Urbach model may still hold in the case of PSI, provided strong coupling existing between its Chl *a* molecules [51]. As shown in [29], in excitonic systems, the vibronic couplings are suppressed, while, in contrast, the electron–phonon couplings may be enhanced. This is especially relevant when charge transfer states strongly coupled to the environment are involved, as arguably is the case in PSI.
